# Characterizing Lymphangiogenesis and Concurrent Inflammation in Adipose Tissue in Response to VEGF-D

**DOI:** 10.3389/fphys.2020.00363

**Published:** 2020-04-22

**Authors:** Adri Chakraborty, Caroline K. Scogin, Kinza Rizwan, Thomas S. Morley, Joseph M. Rutkowski

**Affiliations:** ^1^Division of Lymphatic Biology, Department of Medical Physiology, Texas A&M University College of Medicine, Bryan, TX, United States; ^2^Touchstone Diabetes Center, Department of Internal Medicine, University of Texas Southwestern Medical Center, Dallas, TX, United States

**Keywords:** lymphangiogenesis, VEGFR-3, lymphatic, obesity, VEGF-D, metabolic syndrome

## Abstract

The metabolic consequences of obesity arise from local inflammation within expanding adipose tissue. In pre-clinical studies targeting various inflammatory factors, systemic metabolism can be improved through reduced adipose inflammation. Lymphatic vessels are a critical regulator of inflammation through roles in fluid and macromolecule transport and immune cell trafficking and immunomodulation. Lymphangiogenesis, the expansion of the lymphatic network, is often a necessary step in restoring tissue homeostasis. Using Adipo-VD mice, a model of adipocyte-specific, inducible overexpression of the potent lymphangiogenic factor vascular endothelial growth factor-D (VEGF-D), we previously identified that dense *de novo* adipose lymphatics reduced immune accumulation and improved glucose homeostasis in obesity. On chow diet, however, Adipo-VD mice demonstrated increased adipose tissue immune cells, fibrosis, and inflammation. Here, we characterize the time course of resident macrophage accumulation and lymphangiogenesis in male and female Adipo-VD mice fed chow and high fat diets, examining multiple adipose depots over 4 months. We find that macrophage infiltration occurs early, but resolves with concurrent lymphatic expansion that begins robustly after 1 month of VEGF-D overexpression in white adipose tissue. In obesity, female Adipo-VD mice exhibit reduced lymphangiogenesis and maintain a more glycolytic metabolism compared to Adipo-VD males and their littermates. Adipose lymphatic structures appear to expand by a lymphvasculogenic mechanism involving lymphatic endothelial cell proliferation and organization with a cell source we that failed to identify; hematopoietic cells afford minimal structural contribution. While a net positive effect occurs in Adipo-VD mice, adipose tissue lymphangiogenesis demonstrates a dichotomous, and time-dependent, inflammatory tissue remodeling response.

## Introduction

Tissue inflammation, fibrosis, and immune cell accumulation are all characteristic of adipose tissue expansion in obesity (Sun et al., 2011; Rutkowski et al., 2015). This results in metabolic dysfunction locally, and a spill-over of cytokines and lipids to the system, thus driving the metabolic syndrome (Sun et al., 2013). Genetically altering adipose tissue immune populations, fibrosis, or inflammation has demonstrated efficacy at correcting overall metabolic health (Sun et al., 2011, 2013; Card et al., 2014; Rutkowski et al., 2015). Lymphangiogenesis, the expansion of the lymphatic vessel network, is a common process during tissue inflammation. Lymphatic vessel roles in regulating interstitial fluid balance, macromolecule and immune cell clearance, and the peripheral immune response makes lymphangiogenesis an important part of inflammation resolution in a variety of tissue pathologies (Wiig and Swartz, 2012; Abouelkheir et al., 2017).

The lymphangiogenic proteins vascular endothelial growth factor C (VEGF-C) and –D (VEGF-D), ligands for VEGFR-3, are elevated in adipose tissue during obesity (Karaman et al., 2015; Chakraborty et al., 2019). In humans, lymphatic density varies greatly from adipose depot to depot, but local lymphatic vessel function may still impact local adipose health (Redondo et al., 2020; Varaliova et al., 2020). In mice, lymphatics are absent in brown adipose tissue, rare in gonadal white adipose tissue, and sparse in subcutaneous adipose depots. Tackling the VEGFR-3 signaling axis in obesity has resulted in dichotomous effects on adipose inflammation with some studies worsening and others improving metabolism (Karaman et al., 2015, 2016; Chakraborty et al., 2019). We recently demonstrated that overexpressing VEGF-D specifically in adipose tissue resulted in *de novo* lymphatics in subcutaneous adipose tissues and improved overall metabolism in mice (Chakraborty et al., 2019). The interplay of adipose inflammation and lymphangiogenesis upon elevated VEGFR-3 ligand availability thus remains unclear.

Since lymphatic expansion is naturally absent in murine adipose tissue during obesity, the source of new lymphatic structures upon VEGF-D induction in Adipo-VD mice is unknown. Lineage tracing studies have identified tissue specific venous and non-venous progenitors of lymphatic endothelial cells (LECs) contributing toward lymphatic network expansion during development, particularly in the skin (Srinivasan et al., 2007; Martinez-Corral et al., 2015). In the adult, new lymphatics arise by both sprouting lymphangiogenesis and from several potential progenitor pools, including resident PDGFRβ^+^ cells and infiltrating hematopoietic or immune cells (Maruyama et al., 2005; Klotz et al., 2015; Martinez-Corral et al., 2015; Ulvmar et al., 2016). The mechanisms, and cell source, of adipose lymphatic expansion may provide insight into the potential for these newly present tissue LECs to modulate the immune response.

In this study, we utilized the Adipo-VD mouse with adipose-specific VEGF-D induction under a doxycycline-controllable transgene (Lammoglia et al., 2016; Chakraborty et al., 2019) to identify mechanisms of adipose lymphangiogenesis. We hypothesized that macrophage chemotaxis precedes lymphatic structure formation and that these macrophages may play a role in lymphangiogenesis. Comparison were made between male and female Adipo-VD mouse lymphatic densities and metabolic outcomes on chow and high fat diets and to determine if gender or obesity impacts new lymphatic growth or metabolic response. Lymphangiogenic activity was assessed over time and potential sources of new LECs were tested. This time-course characterization helps elucidate the interplay between lymphatic expansion and inflammation and provides some clues about the potential origins of the cell populations contributing toward induced adipose lymphangiogenesis.

## Materials and Methods

### Animals

Genetic adipose-specific VEGF-D expression in Adipo-VD mice was accomplished as previously described (Lammoglia et al., 2016; Chakraborty et al., 2019). Prox1-tdTomato mice were derived at the Texas Institute for Genomic Medicine by *in vitro* fertilization from sperm obtained from the Mutant Mouse Resource & Research Centers (stock 036531). Mice were backcrossed 4 generations to C57Bl6/J before crossing to C57Bl6/J Adipo-VD mice, then further backcrossed 3-4 generations to C57Bl6/J. Donor constitutive tdTomato mice were made by selecting for Jackson Rosa26STOP-tdTomato mice (stock 007909) demonstrating spontaneous germline excision following a cross with TRE-Cre mice (stock 006234); ubiquitous expression, clearly identified visually, was confirmed genetically by lack of the loxP-flanked neomycin “stop” cassette.

All Adipo-VD mice were used as hemizygous for the TRE-VEGF-D transgene and either wildtype or hemizygous for the *AdipoQ*–rtTA transgene as non-functional and functional mice indicated as –rtTA and +rtTA throughout the text and figures. Age matched male and female mice were used in experiments and housed with 12-h light-dark cycles and ad libitum access to water and food in Association for Assessment and Accreditation of Laboratory Animal Care International. Starting at 6 weeks of age, all mice were fed custom chow diet (Bio-Serv F4107; Bio-Serv, Flemington, NJ) or 60% kcal from fat [D16042102, lard based (supplemented D12492); Research Diets, Inc., New Brunswick, NJ], each containing 600 mg/kg doxycycline to control for its potential effects on –rtTA littermate metabolism. Studies were performed at 1, 2, 3, and 4 months of feeding (mice thus aged up to approximately 24 weeks). Mice raised on standard facility chow were used as a normalization control for qPCR measurements as described. All animal study protocols were approved by the Institutional Animal Care and Use Committee at Texas A&M University (College Station, TX) or UT Southwestern Medical Center (Dallas, TX).

### Tissue and RNA Preparation

Following exsanguination under isoflurane, mouse hair was removed from the mouse inguinal skin area. The entire subcutaneous inguinal white adipose tissue depot (with and without skin attached) along with intrascapular brown adipose depot (surrounding white adipose tissue trimmed), gonadal white adipose, and inguinal white adipose depots were harvested and weighed. Tissues were flash frozen immediately in liquid N_2_ for RNA extraction or fixed in 10% buffered zinc formalin. RNA was extracted using Zymo Direct-zol RNA Miniprep Plus, according to the manufacturer's instructions (Zymo Research, Irvine, CA). Reverse transcription of 1 μg RNA was performed using the iScript cDNA Synthesis kit instructions (Bio-Rad Laboratories, Inc., Hercules, CA).

### Quantitative Real-Time RT-PCR

Adipose tissue cDNAs were amplified using BioRad iTaq universal SYBR Green supermix (Bio-Rad Laboratories, Inc.) in 5 μL reactions on a 384-well 7900T quantitative PCR machine (Applied Biosystems, Foster City, CA). Analyses in adipose tissues utilized *Ubc* as the most reproducible universal control, with expression represented as 2^ΔΔCT^ compared to untreated control mouse adipose RNA. Primer sequences are listed in Table 1.

**Table 1:** Quantitative Real-Time RT-PCR mouse primers utilized for gene expression analysis.

**Target**	**Forward**	**Reverse**
*Cd206*	5′-CAGGTGTGGGCTCAGGTAGT-3′	5′-TGTGGTGAGCTGAAAGGTGA-3′
*F4/80*	5′-CTTTGGCTATGGGCTTCCAGTC-3′	5′-GCAAGGAGGACAGAGTTTATCGTG-3'
*Tgfb*	5′-GGGCCTCTTCTGCGATTTC-3′	5′-ATCCAGGCAAGTGCATTGGTA-3′
*Tnfa*	5′-GAGAAAGTCAACCTCCTCTCTG-3′	5′-GAAGACTCCTCCCAGGTATATG-3′
*Ubc*	5′-GCCCAGTGTTACCACCAAGAAG-3′	5′-GCTCTTTTTAGATACTGTGGTGAGGAA-3'
*Vegfr3*	5′-ATCAGAAGATCGGGCGCTGTTGTA-3′	5′-TGTGTCATGTCCGCCCTTCAGTTA-3′
*Il6*	5′-GAGGATACCACTCCCAACAGACC-3′	5′-AAGTGCATCATCGTTGTTCATA-3′
*Il10*	5′-GCTCTTACTGACTGGCATGAG-3′	5′-CGCAGCTCTAGGAGCATGTG-3′

### Histology and Immunofluorescence

Twenty-four-hours post fixation, tissues were thoroughly rinsed in deionized water and stored in 50% ethanol:water until processed for paraffin embedding. 3-4 μm thick sections were cut to maximum area (lymph node included for inguinal depot). For whole mount imaging, Prox1-tdTomato x Adipo-VD adipose depots were tissue cleared using the PEGASOS immunolabeling and clearing protocol (Jing et al., 2018). Immunofluorescence was detected using single track Zeiss Lightsheet Z1 microscope using an EC Plan-Neofluar 5x/0.16 objective. For widefield immunofluorescence, sections were deparaffinized, rehydrated and labeled for LYVE1, CD11b, tdTomato and Ki-67 with fluorophore- conjugated secondary detection (All antibodies are listed in Table 2). Immunofluorescence was imaged using an Axio-Observer fluorescence microscopy system and MRc camera (Zeiss, Thornwood, NY) or an Eclipse E600 microscope (Nikon, Melville, NY), and images were captured using cellSens Standard version 1.18 imaging software (Olympus, Waltham, MS). Several thick (20–30 μm) sections or hand-trimmed whole mount tissues were prepared and imaged as thin sections, above, but with composite z-stack maximal projections rendered for an in-focus 2D image using a Zeiss Stallion Digital Imaging Workstation or Olympus Fluoview FV3000 Confocal microscope.

**Table 2:** List of primary antibodies along with RRID and catalog number used for immunofluorescence and lightsheet imaging.

**Antigen**	**Source**	**Catalog number**	**RRID**
CD11b	Abcam	ab133357	AB_2650514
Ki-67	Abcam	ab15580	AB_443209
LYVE1	R&D Systems	AF2125	AB_2297188
Mac2	Cedarlane	CL8942AP	AB_10060357
Podoplanin	R&D Systems	AF3244	AB_2268062
VEGFR3	R&D Systems	MAB3491	AB_2105112
tdTomato	Mybiosource	MBS448092	AB_2827808

### Image Analysis

Image analysis of the histology sections was performed using ImageJ software version 1.52 (NIH, Bethesda, MD; http://imagej.nih.gov/ij) to determine the positive immunolabeled areas for LYVE1^+^ and Mac2^+^. For each quantified tissue, four to five random fields were captured at 10X magnification as the tissue area permitted at the same exposure settings for each fluorophore. Larger blood vessel regions and tissue defects were purposefully avoided. In ImageJ, positive labeling was identified by a fluorescence threshold, selected to differentiate lymphatic structures from the background, and measured as area/total area. Images were first averaged per animal, normalized to –rtTA chow-fed controls at 1-month, and then averaged per group. Whole mount images of the adipose were quantified using volume analysis in IMARIS software. The volume of the labeled lymphatics was calculated as [positive volume—(lymph node + afferent/efferent lymph duct volumes)] and reported as the percentage of total image volume.

### Metabolic Measurements

To assess metabolic activity, food intake, respiratory exchange rate (RER), and heat production were measured. Mice were housed for 72 h (24-h acclimatization, 48-h collected measures) in TSE PhenoMaster cages (TSE Systems, Inc., Chesterfield, MO) with recorded ad libitum access to their respective diets and water. Cumulative values are reported for the final 12-h light and dark cycles. Serum glucose levels were measured using colorimetric reagents as before (Chakraborty et al., 2019) following a 4 h fast and 30 min post an intraperitoneal injection of 0.75 U/kg insulin.

### Bone Marrow Transplant

Adipo-VD mice were exposed to 1,400 rads in an Ullman 113, Shepherd Mark I Irradiator. One week prior to irradiation, mice were provided sterilized pH 2.6 drinking water containing 0.1 mg/ml neomycin (Sigma). Bone marrow cells were sterilely isolated from mice constitutively expressing tdTomato and 5 × 10^6^ were delivered intravenously to mice 2 h after irradiation. One mouse per cage was not reconstituted as a lethal irradiation control; these mice died within 1 week. Mice were maintained on acidic water with neomycin for 2 weeks following irradiation then switched to acidic water only for 4 weeks. After 6 weeks of recovery, mice were returned to facility drinking water and started on 600 mg/kg dox-containing chow as above. Mice were terminated following 2 months of VEGF-D induction as above for tissue assessment.

### Statistical Analysis and Data Presentation

All mice carry a single copy of the TRE-VEGF-D transgene (VD). The lack or the presence of the *Adipoq*–rtTA transgene designates Adipo-VD vs. littermates, respectively. Time course studies used 8 vs. 8 mice for histology studies and all subsequent measures started with minimally five vs. five mice per group based on the cage distribution at weaning. Mice were re-genotyped for the rtTA transgene at termination for confirmation, which altered some planned group numbers. Metabolic cages mice ranged from 3–8 mice across the groups. Final group numbers are reported in the Figure Legends for each experiment. Statistical significance for column data was tested by unpaired *t*-test comparisons, assuming unequal variance between the Adipo-VD and littermate groups using GraphPad Prism software version 7.05 (GraphPad Software, La Jolla, CA). For grouped column data multiple comparison two-way ANOVA followed by Holm-Šídák test were performed in GraphPad Prism software. Standard deviation is displayed throughout except standard errors are displayed for grouped graphical time courses for visual clarity. Significance across the +rtTA and –rtTA groups have been represented at ^*^. Significance from month 1 for the subsequent months of each genotype has been represented as #.

## Results

### Lymphatic Expansion and Reduced Crown-Like Structures in Male Adipo-VD Mice

**Figure 1:**
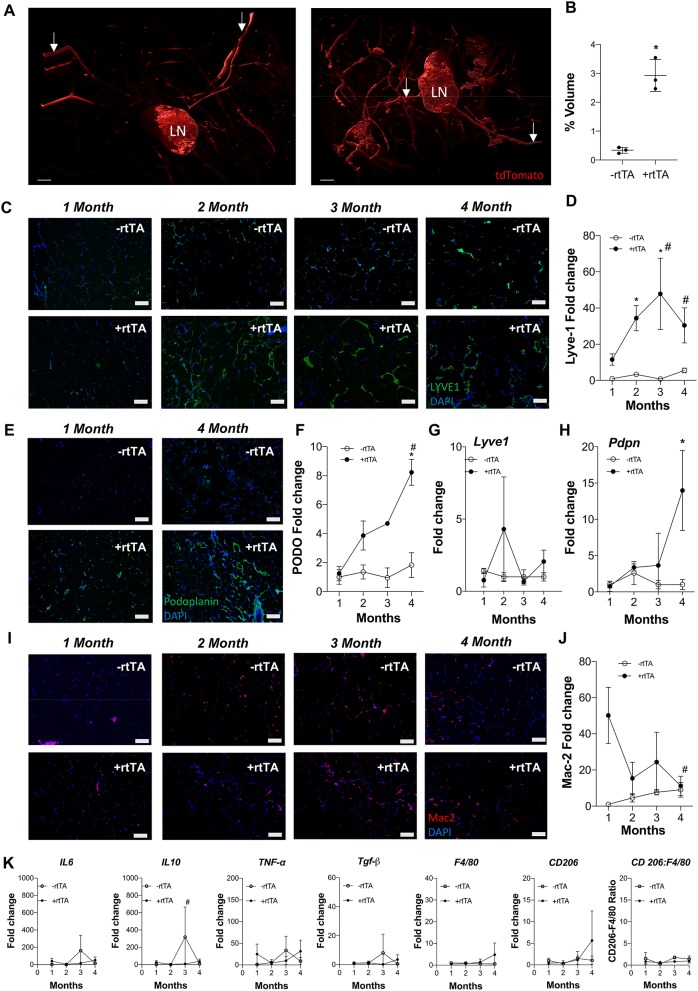
Subcutaneous inguinal adipose lymphatics and inflammation in high fat diet fed male Adipo-VD mice. **(A)** Gradient imaging of subcutaneous adipose lymphatic structures in Adipo-VD x Prox1-tdTomato mice (Red = tdTomato); White arrows: presumed afferent and efferent lymph ducts; lymph node (LN).Scale bar = 500 μm. **(B)** Percentage positive volume of lymphatics within the tissue (LN and lymphatic ducts excluded) **(C)** LYVE1 (green) immunofluorescence of lymphatic structures in male –rtTA and +rtTA subcutaneous inguinal adipose following 1, 2, 3, and 4-month high fat diet feeding. **(D)** LYVE1 pixel area fold change comparison between –rtTA vs. +rtTA mouse inguinal depots quantified from random imaging and all values normalized to –rtTA tissues at 1 month. **(E)** Podoplanin immunofluorescence (green) of lymphatic structures in male –rtTA and +rtTA inguinal adipose depot following 1 and 4-month high fat diet feeding. **(F)** Podoplanin pixel area fold change comparison between –rtTA vs. +rtTA inguinal depot quantified from random imaging and all values normalized to –rtTA tissues at 1 month. **(G,H)** QPCR time course relative expression of *Lyve1* and *Pdpn* between –rtTA and +rtTA inguinal depot normalized to untreated control mouse adipose. **(I)** Macrophage Mac2^+^ crown like structures (red) in –rtTA and +rtTA male subcutaneous inguinal adipose depot following 1, 2, 3, and 4-month high fat diet feeding. **(J)** Mac2+ pixel area fold change comparison between –rtTA vs +rtTA inguinal depot quantified from random imaging and all values normalized to –rtTA tissues at 1 month. **(K)** QPCR immune profile of *IL6, IL10, TNF-*α*, TGF-*β*, CD206, F4/80*, and ratio of *CD206:F4/80*-fold change across the time course normalized to untreated control mouse adipose. Images **(C–I)**, blue = DAPI and scale bars = 20 μm. **(B)** *n* = 3,3 **(D,J)** *n* = 8,8. **(F–H,K)** *n* = 5,5. *P < 0.05 vs. –rtTA; ^#^*P* < 0.05 vs. 1 month.

Adipose expansion is accompanied by obesity-associated inflammation, metabolic dysregulation, and potentially reduced lymphatic density (Nitti et al., 2016; Escobedo and Oliver, 2017). To test the interplay between lymphvasculogenesis and inflammation, Adipo-VD male mice were put on a 60% kcal from fat diet containing 600 mg/kg doxycycline or custom chow diet containing 600 mg/kg doxycycline for 1-4 months. We observed marked lymphatic expansion in the +rtTA inguinal adipose tissue after 4-months of VEGF-D induction (Figure 1A). Three-dimensional rendering of the Prox1-tdTomato+ adipose lymphatic network revealed increased vessels and dense lymphatic regions in +rtTA mice compared to –rtTA mice (Supplementary Videos 1, 2, respectively). Percentage volume quantification of tdTomato positive structures confirmed more lymphatic structures in the +rtTA mice (Figure 1B). LYVE1+ lymphatic area expansion along 1–4 month of HFD feeding demonstrated prolific lymphatic expansion in the white inguinal adipose depots of +rtTA mice (Figure 1C). Comparisons across the –rtTA and +rtTA groups for each month demonstrated significant lymphatic expansion at month 2 &3 for male HFD +rtTA mice compared to –rtTA mice and significantly more LYVE1+ area at 4 months (Figure 1D). Under chow fed conditions, despite the presence of LYVE1+ lymphatic structures early, image quantitation revealed a significant increase in +rtTA white inguinal adipose only at 4 months (Supplementary Figures 1A,B). In both feeding conditions, lymphatic structures were also immunolabeled with podoplanin (Figure 1E, Supplementary Figure 1C). Image quantitation demonstrated a significant increase in podpoplanin+ area under obese conditions at 4 months (Figure 1F), but no significant difference under chow feeding (Supplementary Figure 1D). Quantitative real time PCR was used to confirm these image analyses. On HFD, there was no significant difference in *Lyve1* between groups or over time (Figure 1G), while *Pdpn* expression was significantly elevated in 4 month +rtTA mice, mirroring the image analyses (Figure 1H). Under chow fed conditions, the trends in *Lyve1* and *Pdpn* were similar to the image analysis with no significance identified (Supplementary Figures 1E,F).

Antibodies against Mac2 are used to identify adipose “crown-like structures,” or resident macrophages that surrounding unhealthy adipocytes, in inflamed, obese adipose tissue (Asterholm et al., 2012a; Murano et al., 2013). Mac2+ labeling was visualized (Figure 1I) and quantified by image analysis (Figure 1J) on HFD. Adipo-VD mice (+rtTA) demonstrated significantly higher Mac2+ area early at 1 month compared to their –rtTA littermates, indicating increased crown-like structure formation. This elevation was significantly reduced by month 4, when adipose lymphatics were present (Figure 1J). Mac2+ area in chow feed males appeared to be higher (Supplementary Figure 1G), but their area quantification (Supplementary Figure 1H) was not significant.

While the *AdipoQ* promoter should be active in all adipocytes (Wang et al., 2010), we observed no LYVE1+ lymphatic structures in the epididymal depot of either HFD or chow fed +rtTA mice suggesting that chronic, low-level VEGF-D overexpression is insufficient to expand lymphatics in visceral depots of Adipo-VD mice (Supplementary Figure 2).

Despite increased lymphatics and reduced crown-like structures, qPCR measurement of inflammation-associated genes in +rtTA mice under HFD were largely unchanged compared to –rtTA mice over the times analyzed (Figure 1K). Only *Il10* was significantly reduced at 3 months, likely resulting from an odd increased expression in –rtTA mice. No significant differences were identified between groups or over time for *Il6, Tnfa, Tgfb, Cd206*, or *F480*, nor was the ratio of *Cd206*:*F480* changed (Figure 1K). These null results mirrored our previous work that found that despite reduced immune cell numbers, inflammatory markers were largely equivalent to wildtype mice during HFD-induced obesity (Chakraborty et al., 2019). Under chow feeding, despite early macrophage accumulation gene expression levels of the same markers were not increased in +rtTA Adipo-VD mice and were, surprisingly, less than –rtTA mice for *Il6* and *Il10* at 3 months and *Tnfa* at 4 months (Supplementary Figure 1I). Again, these negative results over time mirrored the lack of differences seen previously at 4 months in this model (Chakraborty et al., 2019).

Lymphangiogenesis was robust in the interscapular brown adipose tissue of +rtTA mice on HFD (Supplementary Figure 3A). Image quantitation revealed this to be highly variable between mice, but significantly increased by 2 months (Supplementary Figure 3B). Chow fed +rtTA mice also demonstrated adipose lymphangiogenesis in interscapular brown adipose as early as 2 months following VEGF-D induction (Supplementary Figure 3C). Image quantitation revealed this to also be highly variable between mice and surprisingly not significant (Supplementary Figure 3D).

In total, male Adipo-VD +rtTA mice exhibited variable, but significant lymphatic expansion in multiple adipose depots within 2 months of VEGF-D overexpression with more profound increases in lymphatic structures measured during HFD feeding.

### Female Adipo-VD Mice Exhibit Adipose Lymphatic Expansion and Reduced Crown-Like Structures

**Figure 2:**
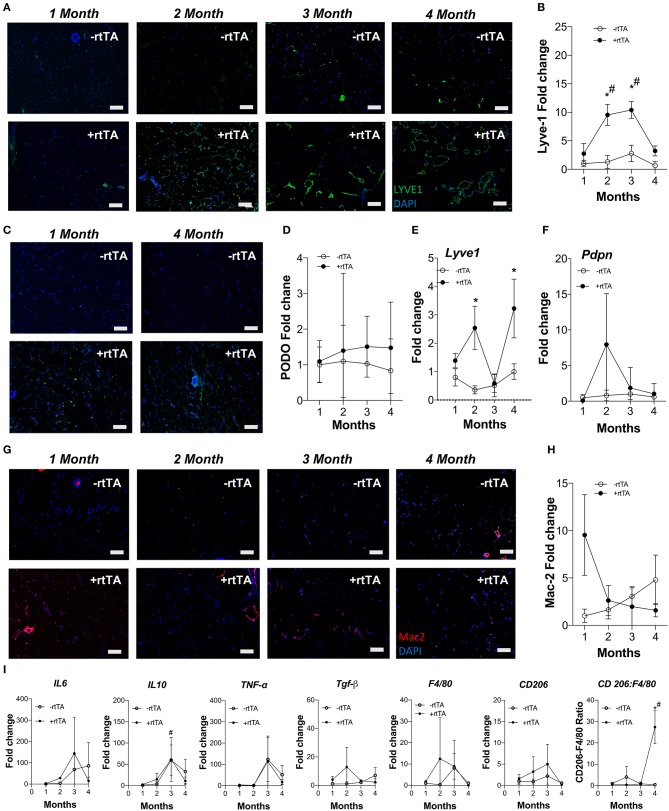
Subcutaneous inguinal adipose lymphatics and inflammation in high fat diet fed female Adipo-VD mice. **(A)** LYVE1 (green) immunofluorescence of lymphatic structures in female –rtTA and +rtTA subcutaneous inguinal adipose following 1, 2, 3, and 4-month high fat diet feeding. **(B)** LYVE1 pixel area fold change comparison between –rtTA vs +rtTA mouse inguinal depots quantified from random imaging and all values normalized to –rtTA tissues at 1 month. **(C)** Podoplanin immunofluorescence (green) of lymphatic structures in female –rtTA and +rtTA inguinal adipose depot following 1 and 4-month high fat diet feeding. **(D)** Podoplanin pixel area fold change comparison between –rtTA vs +rtTA inguinal depot quantified from random imaging and all values normalized to –rtTA tissues at 1 month. **(E,F)** QPCR time course relative expression of *Lyve1* and *Pdpn* between –rtTA and +rtTA inguinal depot normalized to untreated control mouse adipose. **(G)** Macrophage Mac2^+^ crown like structures (red) in –rtTA and +rtTA female subcutaneous inguinal adipose depot following 1, 2, 3, and 4-month high fat diet feeding. **(H)** Mac2+ pixel area fold change comparison between –rtTA vs +rtTA inguinal depot quantified from random imaging and all values normalized to –rtTA tissues at 1 month. **(I)** QPCR immune profile of *IL6, IL10, TNF-*α*, TGF-*β*, CD206, F4/80* and ratio of *CD206:F4/80*-fold change across the time course normalized to untreated control mouse adipose. Images **(A–G)**, blue = DAPI and scale bars = 20 μm. **(B,H)** *n* = 8,8. **(D–F,I)** *n* = 5,5. ^*^P < 0.05 vs. –rtTA; ^#^*P* < 0.05 vs. 1 month.

Females demonstrate an increased propensity for diseases of lymphatic overgrowth, such as lymphangioleiomyomatosis, and lymphatic dysfunction as in lymphedema (Moir, 2016; Morfoisse et al., 2018), yet are protected from the metabolic syndrome in obesity (Karastergiou et al., 2012). We thus sought to test if lymphangiogenesis and inflammation in female Adipo-VD tissues were equivalent to our findings in males. Following a similar protocol and analysis as above, we identified an increase in LYVE1+ lymphatic structures in +rtTA female white inguinal on HFD that was consistent with the males with structures forming by month 2 (Figure 2A). Image quantitation of LYVE1+ pixel area confirmed this with comparisons across the –rtTA and +rtTA groups for each month demonstrating significant lymphatic expansion at months 2 and 3 for female +rtTA mice (Figure 2B). The LYVE1+ pixel area at 2, 3, and 4 months in females was less significantly less than in males (by *post-hoc* analysis; *P* < 0.01, *P* < 0.01, and *P* = 0.048, respectively). While structures were visualized by podoplanin immunolabeling (Figure 2C), no significant difference in podoplanin+ pixels was measured in female HFD inguinal adipose tissue over time (Figure 2D). Under chow diet feeding, while LYVE1+ structures were visually present after 2 months of VEGF-D induction (Supplementary Figure 4A), quantification of LYVE1+ pixel area confirmed a pattern of progressive lymphatic structure expansion only after 4 months of VEGF-D induction (Supplementary Figure 4B). Podoplanin immunolabeling also identified increased structures in female +rtTA inguinal adipose tissue (Supplementary Figure 4C) that was quantified and found to be significant after 4 months (Supplementary Figure 4D). Confirmation of these image analyses by qPCR identified that on HFD, *Lyve1* expression was significantly increased in +rtTA mice at 2 and 4 months (compared to –rtTA) (Figure 2E), while *Pdpn* expression changes were not significant, mirroring the image findings (Figure 2F). Under chow fed conditions, the trends in *Lyve1* and *Pdpn* were highly variable (Supplementary Figures 4E,F).

Adipose depot resident macrophages, “crown like structures,” were identified by Mac2^+^ immunofluorescence in female mice after only 1 month of VEGF-D overexpression in both HFD and chow fed conditions (Figure 2G, Supplementary Figure 4G). Subsequent area quantification demonstrated that on chow diet these areas were significantly reduced from this early peak at 2, 3, and 4 months (Supplementary Figure 4H). This trend was present during HFD, but statistically not significant (Figure 2H).

Similar to the findings in males, gene expression of inflammatory markers *Il6, Il10, Tnfa, Tgfb, Cd206*, and *F480* were mostly unchanged over time and between –rtTA and +rtTA female mice on HFD (Figure 2I). An increased ratio of *Cd206*:*F480*, potentially an indicator of macrophage anti-inflammatory polarization, was significant at 4 months, but would require flow cytometry to confirm. Gene expression in chow feeding largely mirrored the findings in males (Supplementary Figure 4I).

In brown adipose tissue, sporadic expansion of LYVE1+ structures were observed in +rtTA mice as demonstrated by area quantification under both HFD (Supplementary Figures 5A,B) and chow conditions (Supplementary Figures 5C,D). Visually present at 1 month regardless of diet, image analysis demonstrated a significant increase in LYVE1+ structure area at 1 month under chow feeding and 2 months with HFD. In both diets, these trends or significant increases were maintained through 4 months.

Female +rtTA Adipo-VD mice thus exhibit a similar, though somewhat reduced, propensity for adipose tissue lymphangiogenesis compared to males and an early increase in Mac2+ structures in white adipose tissue that significantly resolves over time.

### Metabolic Assays Identify Changes in Male and Female Adipo-VD Mice Across High Fat and Chow Diet Conditions

**Figure 3:**
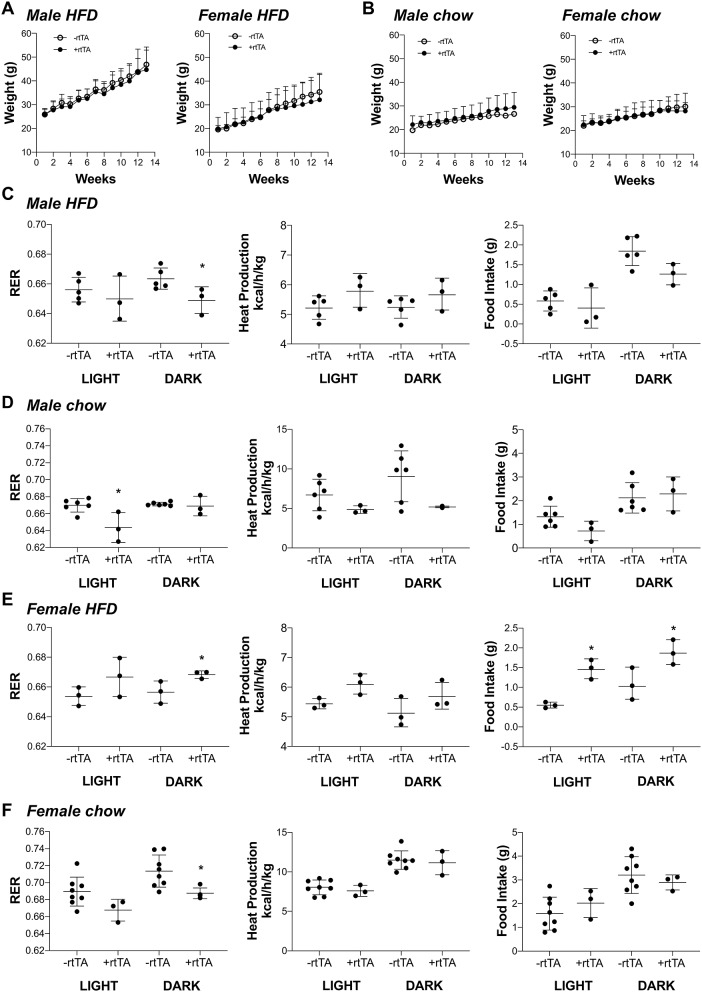
Metabolic analyses of male and female Adipo-VD mice during high fat and chow diet conditions. **(A)** Weight gain curve for high fat diet fed –rtTA and +rtTA male (*n* = 5,3) and female (*n* = 3,3) Adipo-VD mice over 4 months. **(B)** Weight gain curve for chow diet fed –rtTA and +rtTA male (*n* = 6,3) and female (*n* = 8,3) Adipo-VD mice over 4 months. Metabolic cage data were obtained during the light and dark cycles following over 24 h of acclimatization. **(C)** Cumulative average respiratory exchange ratio [RER: VCO_2_ (generation)/VO_2_ (consumption)], average heat production, and cumulative 12-h food intake for high fat diet fed –rtTA and +rtTA male Adipo-VD mice. n=5,3. **(D)** Cumulative average respiratory exchange ratio (RER), average heat production, and cumulative 12-h food intake for chow fed –rtTA and +rtTA male Adipo-VD mice. *n* = 6,3. **(E)** Cumulative average respiratory exchange ratio (RER), average heat production, and cumulative 12-h food intake for high fat diet fed –rtTA and +rtTA female Adipo-VD mice. n=3,3. **(F)** Cumulative average respiratory exchange ratio (RER), average heat production, and cumulative 12-h food intake for chow fed –rtTA and +rtTA female Adipo-VD mice. *n* = 8,3.

We hypothesized that adipose lymphatic expansion was necessary for the metabolic improvements in glucose handling that we previously described (Chakraborty et al., 2019). Weight gain over time for HFD fed male and female mice was equivalent across genotype over time (Figure 3A) as previously reported (Chakraborty et al., 2019). Similarly, no differences between male –rtTA and +rtTA mice weight gain over time was measured (Figure 3B); there was no difference in genotype in females on chow either (Figure 3B). The body weight and adipose tissue depot weight:body weight ratios were largely unchanged between –rtTA and +rtTA male and female mice on both chow and HFD in the final groups studied (Table 3). Only the gonadal white adipose depots increased significantly over time, but did so under both diets. As a rapid test of insulin sensitivity, all mice were fasted for 4 h prior to a bolus of insulin and glucose levels measured 30 min later. No significant differences were identified across the time groups or between genotypes. While this indicated that our mice were metabolically similar, it suggested that 4 months of HFD feeding had not made any group particularly insulin resistant for this study.

**Table 3:** Characteristics of the mice used in this study.

**Months**	**Sex**	**Genotype**	**Mass (g)**	**IWAT:bw (x10^**∧2**^)**	**GWAT:bw (x10^**∧2**^)**	**BAT:bw (x10^**∧2**^)**	**GLUCOSE (MG/Dl)**
**HIGH FAT DIET**							
1	M	–rtTA	47.2 ± 5.4	1.7 ± 0.50	1.2 ± 0.70	0.7 ± 0.10	135.1 ± 41.3
	M	+rtTA	37.3 ± 4.7	0.8 ± 0.30	1.2 ± 0.13	0.7 ± 0.06	84.60 ± 16.1
	F	–rtTA	24.4 ± 5.7	0.9 ± 0.28	1.3 ± 0.12	0.9 ± 0.32	156.2 ± 22.5
	F	+rtTA	24.2 ± 6.0	0.9 ± 0.35	1.3 ± 0.10	0.8 ± 0.10	141.5 ± 22.9
2	M	–rtTA	35.8 ± 5.8	0.8 ± 0.20	1.7 ± 0.37^**#**^	0.1 ± 0.05	117.5 ± 30.9
	M	+rtTA	30.7 ± 1.8	1.3 ± 0.40	1.3 ± 0.08	0.9 ± 0.10	94.60 ± 28.5
	F	–rtTA	31.0 ± 5.9	1.0 ± 0.10	1.3 ± 0.13	0.8 ± 0.60	88.00 ± 2.40
	F	+rtTA	35.6 ± 2.4	0.7 ± 0.07	1.5 ± 0.42	1.0 ± 0.60	99.50 ± 14.2
3	M	–rtTA	48.8 ± 3.1	2.8 ± 0.10	1.7 ± 0.01^**#**^	1.6 ± 0.23*^#^	172.0 ± 43.3
	M	+rtTA	54.2 ± 1.7	2.0 ± 0.70	1.6 ± 0.03	0.9 ± 0.32	193.5 ± 19.1
	F	–rtTA	29.6 ± 4.7	0.3 ± 0.05	1.4 ± 0.30	0.9 ± 0.02	94.60 ± 4.90
	F	+rtTA	28.4 ± 4.3	0.9 ± 0.30	1.4 ± 0.20	0.9 ± 0.40	117.6 ± 17.8
4	M	–rtTA	42.4 ± 4.7	1.7 ± 0.80	1.7 ± 0.70^**#**^	1.6 ± 0.10^**#**^	98.00 ± 21.1
	M	+rtTA	43.6 ± 8.1*	1.5 ± 0.50	1.6 ± 0.12	1.3 ± 0.32^**#**^	88.20 ± 1.70
	F	–rtTA	35.4 ± 5.4	1.6 ± 0.10	1.9 ± 0.20^**#**^	1.3 ± 0.45	97.30 ± 11.4
	F	+rtTA	33.3 ± 7.6	0.9 ± 0.20	1.9 ± 0.09^**#**^	1.3 ± 0.44	86.80 ± 44.5
**CHOW DIET**							
1	M	–rtTA	32.2 ± 5.2	1.2 ± 1.1	1.1 ± 0.44	1.3 ± 0.13	144.1 ± 28.5
	M	+rtTA	34.2 ± 0.9	0.9 ± 0.04	1.3 ± 0.60	0.9 ± 0.30	117.3 ± 30.9
	F	–rtTA	21.4 ± 1.6	0.5 ± 0.10	1.1 ± 0.86	0.8 ± 0.30	118.2 ± 19.6
	F	+rtTA	22.2 ± 0.6	0.5 ± 0.19	1.3 ± 0.08	1.1 ± 0.11	116.2 ± 12.6
2	M	–rtTA	43.4 ± 10	1.2 ± 0.30	1.3 ± 0.09	0.9 ± 0.2.1	104.4 ± 21.3
	M	+rtTA	36.6 ± 17	1.5 ± 0.01	1.4 ± 0.77	0.1 ± 0.30	123.6 ± 23.7
	F	–rtTA	21.2 ± 2.4	0.5 ± 0.20	1.2 ± 0.03	0.8 ± 0.02	87.20 ± 25.9
	F	+rtTA	23.0 ± 1.4	0.6 ± 0.20	1.4 ± 0.1.1	0.9 ± 0.04	93.20 ± 22.7
3	M	–rtTA	34.2 ± 3.7	0.6 ± 0.05	1.7 ± 0.40^**#**^	1.2 ± 0.01	129.6 ± 15.9
	M	+rtTA	34.2 ± 5.5	1.1 ± 0.34	1.4 ± 0.60	0.1 ± 0.12	142.6 ± 31.4
	F	–rtTA	22.3 ± 2.6	0.5 ± 0.20	1.5 ± 0.01	0.7 ± 0.43	93.40 ± 27.7
	F	+rtTA	23.9 ± 3.4	0.6 ± 0.20	1.4 ± 0.02	1.1 ± 0.90	101.8 ± 18.2
4	M	–rtTA	22.1 ± 2.3	1.4 ± 0.76	1.8 ± 0.30^**#**^	1.3 ± 0.20	126.3 ± 4.10
	M	+rtTA	27.6 ± 6.1	1.4 ± 0.70	1.5 ± 0.01*	1.0 ± 0.12	136.3 ± 2.50
	F	–rtTA	28.2 ± 5.0	1.0 ± 0.60	1.5 ± 0.20	0.7 ± 0.40	94.90 ± 19.0
	F	+rtTA	29.1 ± 3.9	1.4 ± 0.20*	1.6 ± 0.20	1.2 ± 0.32	107.6 ± 27.4

Values represent the mean ± SD. IWAT, inguinal white adipose tissue; GWAT, gonadal white adipose tissue; BAT, brown adipose tissue; bw, body weight. Glucose levels were measured in the fasted state, 30 min following insulin injection. *indicates significant difference between genotypes, # indicates significant difference from 1-month.

To identify potentially more subtle metabolic differences, mice were housed and monitored in Phenomaster metabolic cages. Adipo-VD male mice on a HFD demonstrated significantly reduced respiratory exchange (RER: VCO_2_/VO_2_) during the dark cycle (Figure 3C), suggesting increased fatty acid utilization, with no change in heat production or food intake. During chow feeding, +rtTA Adipo-VD mice also demonstrated a lower RER, though during the day cycle (Figure 3D). Heat production and food intake were again unchanged. Conversely, HFD female +rtTA Adipo-VD mice demonstrated a significantly elevated RER during the dark cycle and ate significantly more food throughout the day (Figure 3E) suggesting increased carbohydrate utilization. Interestingly, under chow feeding, +rtTA females demonstrated a reduced dark cycle RER, like their male counterparts, and heat production and food intake were not significantly different (Figure 3F). In total, the mice used in this study were largely similar in their basic body phenotype and metabolism, with Adipo-VD mice generally utilizing more oxidative respiration than –rtTA mice.

### Lymphatic Proliferation and Lymphvasculogenesis in Adipose Tissue of Adipo-VD Mice

**Figure 4:**
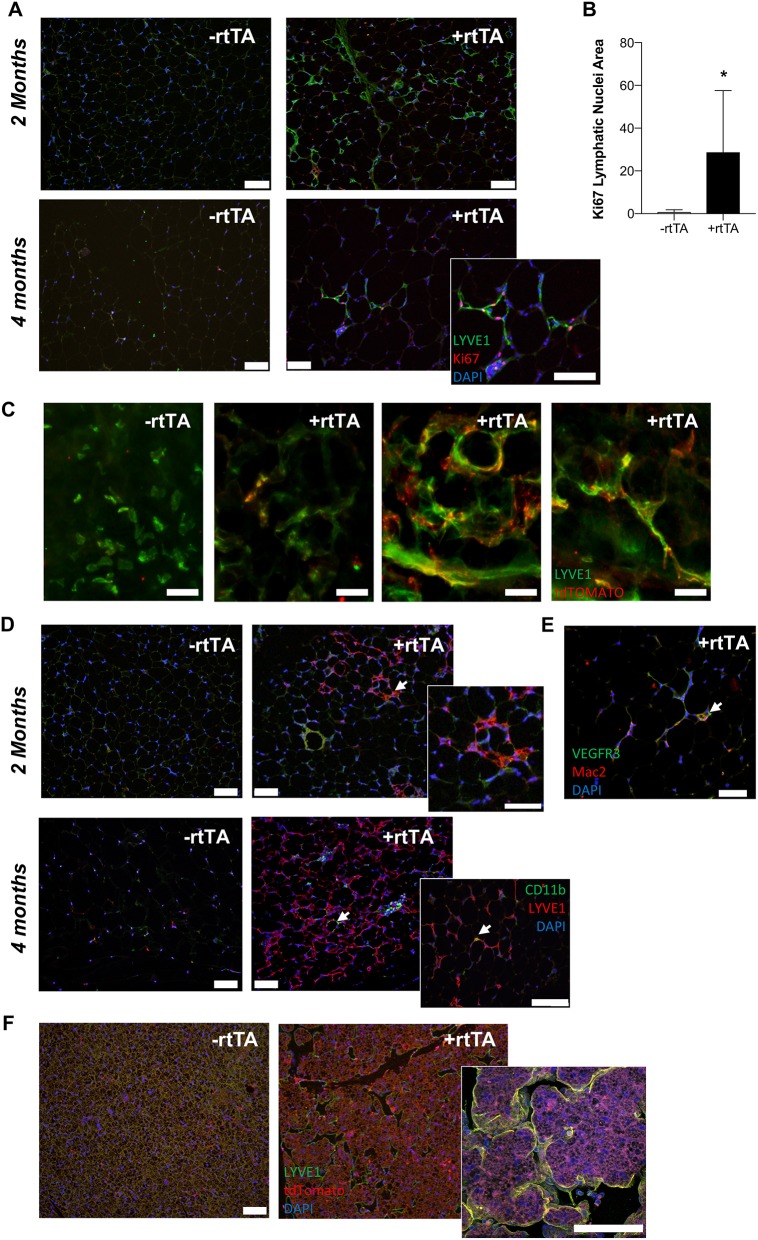
VEGF-D induces LEC proliferation within the inguinal adipose depot. **(A)** Month 2 and 4 immunolabeling of proliferating (Ki-67, red) lymphatics (LYVE1, green) in the inguinal adipose depot of chow fed male –rtTA and +rtTA mice. Blue = DAPI and scale bars = 20 μm. **(B)** Number of Ki-67 positive lymphatic nuclei per 4 cm^2^ section of subcutaneous inguinal adipose of male and female mice. *n* = 5, 5. **(C)** Whole mount images of LYVE1^+^ (green) and Prox1-tdTomato (red) cells and structures in –rtTA and several +rtTA Adipo-VD mouse subcutaneous adipose tissues at 4 months. Scale bars = 50 μm. **(D)** Month 2 and 4 immunofluorescence for lymphatics (LYVE1, red) and CD11b^+^ macrophages (green) in inguinal adipose of –rtTA and +rtTA mice at 2 months. Arrows = potential interaction. Blue = DAPI and scale bars = 20 μm. **(E)** Some Mac2 crown-like structure macrophages (red) can be found to be VEGFR-3 (green) positive (arrow). Blue = DAPI and scale bars = 20 μm. **(F)** Immunofluorescence of LYVE1 (green) and Prox1-tdTomato (red) bone marrow-derived cell expression in –rtTA and +rtTA brown adipose tissues at 2 months. ^*^P < 0.05 vs. –rtTA.

Proliferation, migration, and organization of LECs via VEGFR-3 signaling is part of the lymphangiogenic process (Makinen et al., 2001; Goldman et al., 2007; Karpanen and Alitalo, 2008). When adipocyte VEGF-D expression was induced in high fat diet fed mice, Adipo-VD mice demonstrated lymphatic structure expansion in subcutaneous inguinal adipose tissue at 2 and 4 months (Figure 4A). However, few lymphatics in the adipose tissue were positive for the proliferation marker Ki67 (Figure 4A). At 2 months, Ki-67+ cells were limited to the adjacent dermis (not shown). At 4 months, however, lymphatic structures in the adipose tissue were found to contain Ki67+ cells. The number of Ki-67+ LYVE1+ cells was significantly greater on sections of +rtTA compared to –rtTA tissues (though few lymphatic structures were identified in –rtTA mice), indicative of continued proliferation with chronic VEGF-D overexpression (Figure 4B). Lack of proliferative markers earlier at 2 months suggests inclusion of LECs that may have proliferated elsewhere or earlier or other cell types that newly express LEC markers.

We crossed Prox1-tdTomato mice with Adipo-VD mice to visualize expanded lymphatics in adipose tissue. As demonstrated above, a Prox1-tdTomato+ network of lymphatic structures forms (Figure 1A) in Adipo-VD mice. We identified many LYVE1-expressing cells and cell clusters in thick sections of 2-month adipose tissues of –rtTA and +rtTA mice that were Prox1-tdTomato negative (Figure 4C). These cells have been previously reported by others as macrophages in adipose tissues (Cho et al., 2014). In +rtTA mice, we found that new lymphatic structures demonstrated varying degrees of tdTomato and LYVE1 immunolabeling, co-localized in some places, but independent in others (Figure 4C). Organization of these structures, and lack of early proliferation, suggests a lymphvasculogenic mechanism as previously reported (Chakraborty et al., 2019).

Macrophages reportedly contribute to lymphatic vessel structure in corneal inflammation (Maruyama et al., 2005). In the adipose tissue, this does not appear to occur as CD11b^+^ cells were identified independent of LYVE1+ structures at month 2 of VEGF-D induction, and rarely associate with lymphatic structures at month 4 (Figure 4D). Mac-2+ crown-like structure macrophages were sometimes VEGFR-3+ (Figure 4E). To test the contribution of hematopoietic cells to lymphvasculogenesis, irradiated Adipo-VD mice were reconstituted with constitutive Rosa26-tdTomato bone marrow. Interestingly, lymphangiogenesis did not occur in the inguinal adipose tissue of these mice after 2 months of VEGF-D overexpression; tdTomato+ cells were identified in the lymph node and throughout white and brown adipose (not shown, Figure 4F). The only lymphatic structures found were identified in the brown adipose tissue of one mouse. In that tissue several tdTomato+ cells were identified near lymphatic structures, but did not appear to make any significant structural contribution to the vessels (Figure 4F). Absent further lineage tracing, the precise cell source of expanding adipose lymphatics remains unclear. Lymphatic proliferation and potentially other non-hematopoietic cell types may therefore contribute toward lymphvasculogenesis in Adipo-VD mouse adipose tissues.

### Discussion

Obesity presents a chronic, inflammatory remodeling process in adipose tissue. Inflammation-associated lymphangiogenesis is often necessary to ameliorate inflammation, but despite increased expression of VEGF-C and VEGF-D reported in obesity, lymphatic vessels remain sparse in adipose tissue and those present demonstrate reduced function (Arngrim et al., 2013; Redondo et al., 2020). In this study we demonstrate that locally increasing VEGF-D levels specifically in the adipose tissue of Adipo-VD mice augmented lymphatic vessel structure formation in subcutaneous adipose depots. Obesity does not impair this expansion and male mice demonstrated greater lymphatic densities than females. Despite increased numbers of Mac2+ crown-like structures present in Adipo-VD inguinal adipose depots early, these are significantly reduced over time. Male Adipo-VD mice exhibit a more oxidative metabolism, while female Adipo-VD mice are more glycolytic in obesity than their respective –rtTA littermates. Finally, we identified that the lymphvasculogenesis process of new lymphatic structures in Adipo-VD adipose tissue includes LEC proliferation and other cells of unconfirmed identity.

Lymphatic vessels and adiposity have an intimate relationship. Dysfunctional lymphatics may cause adipose tissue expansion and obesity; conversely, conditions of expanding adipose tissue—obesity, lymphedema, and lipedema—appear to inhibit lymphatic function (Lim et al., 2009; Garcia Nores et al., 2016; Gousopoulos et al., 2017; Al-Ghadban et al., 2019; Gasheva et al., 2019). Reduced lymphatic density has also reported in obese mice (Garcia Nores et al., 2016). In studying the time course of lymphangiogenesis in Adipo-VD mice, we have found that high fat diet feeding and obesity do not impair lymphatic growth. Lymhpangiogenesis occurs quickly and is visually present at 1 month and quantitatively significant at 2 months in obese Adipo-VD mice, faster than the 4 months it required under chow conditions. It is possible that obesity simply does not slow an already rampant process, with induced VEGF-D synergizing with already elevated VEGFR-3 ligands, rather than inhibiting existing lymphatics once truly obese. VEGFR-3 signaling may also improve downstream collecting lymphatic vessel contractility (Breslin et al., 2007), or help to maintain progenitor cells in a lymphatic-like Prox-1+VEGFR-3+ phenotype (Srinivasan et al., 2014), potentially increasing lymphatic function and enhancing lymphangiogenesis in Adipo-VD adipose tissues. Lack of noticeable lymphangiogenesis in the gonadal adipose depot is surprising. The adiponectin promoter is active there with a demonstrated ability to overexpress VEGF-C and VEGF-D (Nitschke et al., 2017; Chakraborty et al., 2019). The depot, particularly in obesity, may demonstrate lower promoter activity as adiponectin levels decline (Sun et al., 2014) or the adult depot may harbor fewer of the cell pools that contribute to induced lymphangiogenesis discussed later. Higher levels or earlier VEGF-D expression, when the tissue is first forming, may still promote visceral lymphangiogenesis.

Adipose tissue inflammation is a hallmark of the metabolic syndrome. Murine studies targeting various immune cell, inflammatory cytokines, fibrotic matrix components, or the vasculature have demonstrated that by limiting the inflammatory response in adipose tissue, systemic health may be protected (Rutkowski et al., 2015). Previous studies manipulating VEGFR-3 signaling in obesity have demonstrated that mice overexpressing VEGF-C in the skin have impaired metabolism in obesity and that systemic VEGFR-3 blockade reduces VEGFR-3+ pro-inflammatory M1 macrophage numbers in adipose tissue (Karaman et al., 2015, 2016). In both male and female Adipo-VD mice, under both chow and high fat diet feeding, adipose VEGF-D induction significantly increases macrophage numbers, judged by Mac2 immunolabeling. This accumulation at 1 month, however, was reduced over time, coinciding with significant lymphatic expansion in each condition. We previously reported that increasing lymphatics in adipose tissue increased immune cell migration from the inguinal subcutaneous adipose tissue to the inguinal lymph node (Chakraborty et al., 2019), so these macrophages may migrate away. Alternatively, as Mac2 is indicative of adipose crown-like structures that form around dysfunctional adipocytes, improved adipose tissue health may necessitate fewer of these cells. The inflammatory markers tested were largely unchanged between –rtTA and +rtTA Adipo-VD mice. Few differences between –rtTA and +rtTA mice in protein products of these genes was reported previously (Chakraborty et al., 2019), but to find no elevation over time in obesity is surprising. It could be that these mice never fully developed the metabolic syndrome or that these markers were measured in the metabolically responsive inguinal depot that saw little increase in mass over time; therefore it is possible inflammatory genes were more upregulated in the gonadal depot that demonstrated the most weight gain in obesity.

Local lymphatic endothelium may serve as an immunomodulatory site, dampening the immune response within the tissue through innate and acquired mechanisms that were not examined in this study (Maisel et al., 2017; Lane et al., 2018). It is also possible that dense lymphatics in Adipo-VD adipose may act like fat associated lymphoid clusters (FALCs) in the tissue (Benezech et al., 2015; Camell et al., 2019). Resident B cells in aging mouse FALCs were recently identified to be negative regulators of adipose health compared to splenic B cells (Camell et al., 2019). While a significant increase in B cell recruitment was previously identified in Adipo-VD adipose (Chakraborty et al., 2019), we failed to identify any co-localization of larger number of B220+ cells with new lymphatic structures by immunofluorescence (not shown); increased B cells may still play a role in the tissue. Obesity also reduces lymph node tissue structure, which may be prevented with lymphangiogenic signaling in Adipo-VD mice (Solt et al., 2019). Our study and others' demonstrate that elevated VEGF-D is likely chemotactic toward pro-inflammatory cells, but chronic overexpression in Adipo-VD mice reduces immune accumulation through several potential mechanisms.

Following the same VEGF-D induction protocol, we previously demonstrated the Adipo-VD mice are protected from obesity's metabolic syndrome, with improved glucose handling and reduced liver lipid deposition (Chakraborty et al., 2019). The mice used in this timecourse characterization were largely equivalent across genotype from a body weight, adiposity, and fasting glucose level. While HFD fed mice did gain more weight than those fed on chow they were not overly obese (averaging <50 g), which makes identifying changes in adipose inflammation and the metabolic syndrome more challenging. Both male and female Adipo-VD mice were more oxidative, judged by a lower RER, on chow diet than their littermates. Males maintained this difference on HFD, while females were more glycolytic. This could be due to overall “healthier” adipose tissue. An alternative interpretation of this data may be how readily Adipo-VD mice adapt to a different fuel source: RER should be reduced once HFD is introduced (Asterholm et al., 2012b). Female Adipo-VD RER was low at ~0.67 under both feeding conditions. Adipo-VD mice may thus be primed for fatty acid utilization in contrast to VEGF-D knockout mice that exhibit higher lipid levels (Tirronen et al., 2018). The roles of lymphatics in lipid utilization are an active area of study in the field.

Both the sprouting of pre-existing lymphatic vessels and circulating transdifferentiating cells have been identified to contribute toward late developmental and adult lymphangiogenesis. Several studies found that non-venous cells, or non-LECs, contribute to lymphatic expansion in the dermis, heart, and mesentery (Ulvmar and Makinen, 2016). Several potential cell sources were proposed in these studies, including circulating angioblasts or hemogenic endothelial cells (Klotz et al., 2015; Martinez-Corral et al., 2015; Stanczuk et al., 2015). Hematopoietic, Vav1^+^/Tie2- cells contributed to cardiac lymphatics (Martinez-Corral et al., 2015). Past studies in the cornea identified CD11b^+^ macrophages contributing to new lymphatics (Maruyama et al., 2005). Hyperplasia of existing vessels and sprouting lymphangiogenesis was previously reported with high levels of VEGF-C overexpression in adipose (Nitschke et al., 2017). Characterization of lymphatics in healing mouse hearts identified two separate mechanisms or sub-populations of cells, sprouting lymphangiogenesis and isolated LECs, contributing toward expanding lymphatics; the cell source of the new LECs was unclear (Gancz et al., 2019). Recently, an imaging study identified LYVE1+ cells from both endothelial and hematopoietic lineage within human adipose tissue (Redondo et al., 2020). This study reinforced that hematopoietic LYVE1+CD45+ cells associate with adipose lymphatics, but they do not form their structure. While LYVE1+ adipose macrophages have been previously implicated in angiogenesis (Cho et al., 2007), our data does not confirm their role in lymphangiogenesis. Rather, we identified proliferation of tissue LECs (once present) and a lymphvasculogenesis mechanism that includes potentially LYVE1^+^ Prox1^−^ cell clusters, but largely excluded cells of hematopoietic origin. Interestingly, lymphatics failed to populate Adipo-VD white adipose tissue following irradiation suggesting that the cell source may have been lost or transformed. Bone marrow adipocytes survive irradiation and do have an impact on the hematopoietic microenvironment (Naveiras et al., 2009): it is possible that expressing VEGF-D during a period of reconstitution changes the marrow populations or their differentiation. There are also tissue-resident and non-hematopoietic endothelial cell precursor pools that demonstrate importance in adipose tissue vascularization (Gavin et al., 2016; Panina et al., 2018). More rigorous lineage tracing experiments, potentially targeting macrophages or PDGFβ+ cells (Ulvmar et al., 2016), would be necessary to truly identify these cells. These experiments in Adipo-VD mice may provide future understanding of the adult lymphvasculogensis process and these new vessels' immunomodulatory functions.

In conclusion, overexpression of VEGF-D in adipose tissue induces macrophage crown-like structure formation and a lymphvasculogenesis process of increased lymphatic vessel density. Increased lymphatics reduce the number of resident adipose tissue macrophages over time and increase systemic fatty acid utilization. The results of local VEGFR-3 activation within adipose tissue thus represent a dichotomous, and time-dependent inflammatory tissue remodeling response.

## Data Availability Statement

The datasets generated for this study are available on request to the corresponding author.

## Ethics Statement

The animal study was reviewed and approved by the Institutional Animal Care and Use Committee at Texas A&M University (College Station, TX) or UT Southwestern Medical Center (Dallas, TX).

## Author Contributions

AC, CS, KR, and JR performed experiments and analyzed data. TM assisted with lineage tracing. AC and JR prepared the manuscript. All authors have read and approve the work.

## Conflict of Interest

The authors declare that the research was conducted in the absence of any commercial or financial relationships that could be construed as a potential conflict of interest.

## Supplementary Material

Supplementary Figure 1 Subcutaneous inguinal adipose lymphatics and inflammation in chow fed male Adipo-VD mice. **(A)** LYVE1 (green) immunofluorescence of lymphatic structures in male –rtTA and +rtTA subcutaneous inguinal adipose following 1, 2, 3, and 4-month chow diet feeding. **(B)** LYVE1 pixel area fold change comparison between –rtTA vs. +rtTA mouse inguinal depots quantified from random imaging and all values normalized to –rtTA tissues at 1 month. **(C)** Podoplanin immunofluorescence (green) of lymphatic structures in male –rtTA and +rtTA inguinal adipose depot following 1 and 4-month chow diet feeding. **(D)** Podoplanin pixel area fold change comparison between –rtTA vs +rtTA inguinal depot quantified from random imaging and all values normalized to –rtTA tissues at 1 month. **(E,F)** QPCR time course relative expression of *Lyve1* and *Pdpn* between –rtTA and +rtTA inguinal depot normalized to untreated control mouse adipose. **(G)** Macrophage Mac2^+^ crown like structures (red) in –rtTA and +rtTA male subcutaneous inguinal adipose depot following 1, 2, 3, and 4-month chow diet feeding. **(H)** Mac2+ pixel area fold change comparison between –rtTA vs +rtTA inguinal depot quantified from random imaging and all values normalized to –rtTA tissues at 1 month. **(I)** QPCR immune profile of *IL6, IL10, TNF-*α*, TGF-*β*, CD206, F4/80*, and ratio of *CD206:F4/80*-fold change across the time course normalized to untreated control mouse adipose. Images **(A–G)**, blue = DAPI and scale bars = 20 μm. **(B,H)** *n* = 8,8. **(D–F,I)** *n* = 5,5. ^*^*P* < 0.05 vs. –rtTA; #*P* < 0.05 vs. 1 month.

Supplementary Figure 2 Gonadal adipose tissue lymphatics and crown-like structures. Representative images of LYVE1 (green) and Mac2 (red) immunofluorescence find no lymphatics within the gonadal adipose tissue depot of chow and HFD –rtTA and +rtTA mice. Blue = DAPI and scale bars = 20 μm.

Supplementary Figure 3 Interscapular brown adipose lymphatic expansion in high fat diet and chow fed male Adipo-VD mice. **(A)** LYVE1 (green) immunofluorescence of lymphatic structures in male –rtTA and +rtTA interscapular brown adipose tissue following 1, 2, 3, and 4-month high fat diet feeding. **(B)** LYVE1 pixel area fold change comparison between high fat diet fed –rtTA vs +rtTA mouse brown adipose depots quantified from random imaging and all values normalized to –rtTA tissues at 1 month. **(C)** LYVE1 (green) immunofluorescence of lymphatic structures in male –rtTA and +rtTA interscapular brown adipose tissue following 1, 2, 3, and 4-month chow diet feeding. **(D)** LYVE1 pixel area fold change comparison between chow fed –rtTA vs +rtTA mouse brown adipose depots quantified from random imaging and all values normalized to –rtTA tissues at 1 month. Images **(A,C)** blue = DAPI and scale bars = 20 μm **(B,D)** *n* = 8. ^*^*P* < 0.05 vs. –rtTA.

Supplementary Figure 4 Subcutaneous inguinal adipose lymphatics and inflammation in chow fed female Adipo-VD mice. **(A)** LYVE1 (green) immunofluorescence of lymphatic structures in female –rtTA and +rtTA subcutaneous inguinal adipose following 1, 2, 3, and 4-month chow diet feeding. **(B)** LYVE1 pixel area fold change comparison between –rtTA vs +rtTA mouse inguinal depots quantified from random imaging and all values normalized to –rtTA tissues at 1 month. **(C)** Podoplanin immunofluorescence (green) of lymphatic structures in female –rtTA and +rtTA inguinal adipose depot following 1 and 4-month chow diet feeding. **(D)** Podoplanin pixel area fold change comparison between –rtTA vs +rtTA inguinal depot quantified from random imaging and all values normalized to –rtTA tissues at 1 month. **(E,F)** QPCR time course relative expression of *Lyve1* and *Pdpn* between –rtTA and +rtTA inguinal depot normalized to untreated control mouse adipose. **(G)** Macrophage Mac2^+^ crown like structures (red) in –rtTA and +rtTA female subcutaneous inguinal adipose depot following 1, 2, 3, and 4-month chow diet feeding. **(H)** Mac2+ pixel area fold change comparison between –rtTA vs +rtTA inguinal depot quantified from random imaging and all values normalized to –rtTA tissues at 1 month. **(I)** QPCR immune profile of *IL6, IL10, TNF-*α*, TGF-*β*, CD206, F4/80*, and ratio of *CD206:F4/80*-fold change across the time course normalized to untreated control mouse adipose. Images **(A–G)**, blue = DAPI and scale bars = 20 μm. **(B,H)** *n* = 8,8. **(D–F,I)** *n* = 5,5. ^*^*P* < 0.05 vs. –rtTA; #*P* < 0.05 vs. 1 month.

Supplementary Figure 5 Interscapular brown adipose lymphatic expansion in high fat diet and chow fed female Adipo-VD mice. **(A)** LYVE1 (green) immunofluorescence of lymphatic structures in female –rtTA and +rtTA interscapular brown adipose tissue following 1, 2, 3, and 4-month high fat diet feeding. **(B)** LYVE1 pixel area fold change comparison between high fat diet fed –rtTA vs. +rtTA mouse brown adipose depots quantified from random imaging and all values normalized to –rtTA tissues at 1 month. **(C)** LYVE1 (green) immunofluorescence of lymphatic structures in female –rtTA and +rtTA interscapular brown adipose tissue following 1, 2, 3, and 4-month chow diet feeding. **(D)** LYVE1 pixel area fold change comparison between chow fed –rtTA vs +rtTA mouse brown adipose depots quantified from random imaging and all values normalized to –rtTA tissues at 1 month. Images **(A,C)** blue = DAPI and scale bars = 20 μm **(B,D)** *n* = 8. ^*^*P* < 0.05 vs. –rtTA.

Supplementary Video 1 Three-dimensional rendering of –rtTA mouse inguinal adipose tissue lymphatics. Prox1-tdTomato+ lymphatic vessels immunolabeled in –rtTA inguinal adipose with an anti-tdTomato antibody then visualized by light sheet microscopy following PEGASOS tissue clearing.

Supplementary Video 2 Three-dimensional rendering of +rtTA mouse inguinal adipose tissue lymphatics. Prox1-tdTomato+ lymphatic vessels immunolabeled in +rtTA Adipo-VD inguinal adipose with an anti-tdTomato antibody then visualized by light sheet microscopy following PEGASOS tissue clearing.
