# Supplementary material for: Characterizing Lymphangiogenesis and Concurrent Inflammation in Adipose Tissue in Response to VEGF-D
Source: Front Physiol. 2020 Apr 22;11:363. doi: 10.3389/fphys.2020.00363 (PMC7188984; doi:10.3389/fphys.2020.00363)
Supplement: Supplementary Figure 1 — Subcutaneous inguinal adipose lymphatics and inflammation in chow fed male Adipo-VD mice. (A) LYVE1 (green) immunofluorescence of lymphatic structures in male –rtTA and +rtTA subcutaneous inguinal adipose following 1, 2, 3, and 4-month chow diet feeding. (B) LYVE1 pixel area fold change comparison between –rtTA vs. +rtTA mouse inguinal depots quantified from random imaging and all values normalized to –rtTA tissues at 1 month. (C) Podoplanin immunofluorescence (green) of lymphatic structures in male –rtTA and +rtTA inguinal adipose depot following 1 and 4-month chow diet feeding. (D) Podoplanin pixel area fold change comparison between –rtTA vs +rtTA inguinal depot quantified from random imaging and all values normalized to –rtTA tissues at 1 month. (E,F) QPCR time course relative expression of Lyve1 and Pdpn between –rtTA and +rtTA inguinal depot normalized to untreated control mouse adipose. (G) Macrophage Mac2+ crown like structures (red) in –rtTA and +rtTA male subcutaneous inguinal adipose depot following 1, 2, 3, and 4-month chow diet feeding. (H) Mac2+ pixel area fold change comparison between –rtTA vs +rtTA inguinal depot quantified from random imaging and all values normalized to –rtTA tissues at 1 month. (I) QPCR immune profile of IL6, IL10, TNF-α, TGF-β, CD206, F4/80, and ratio of CD206:F4/80-fold change across the time course normalized to untreated control mouse adipose. Images (A–G), blue = DAPI and scale bars = 20 μm. (B,H) n = 8,8. (D–F,I) n = 5,5. *P < 0.05 vs. –rtTA; #P < 0.05 vs. 1 month. [file Data_Sheet_1.PDF]

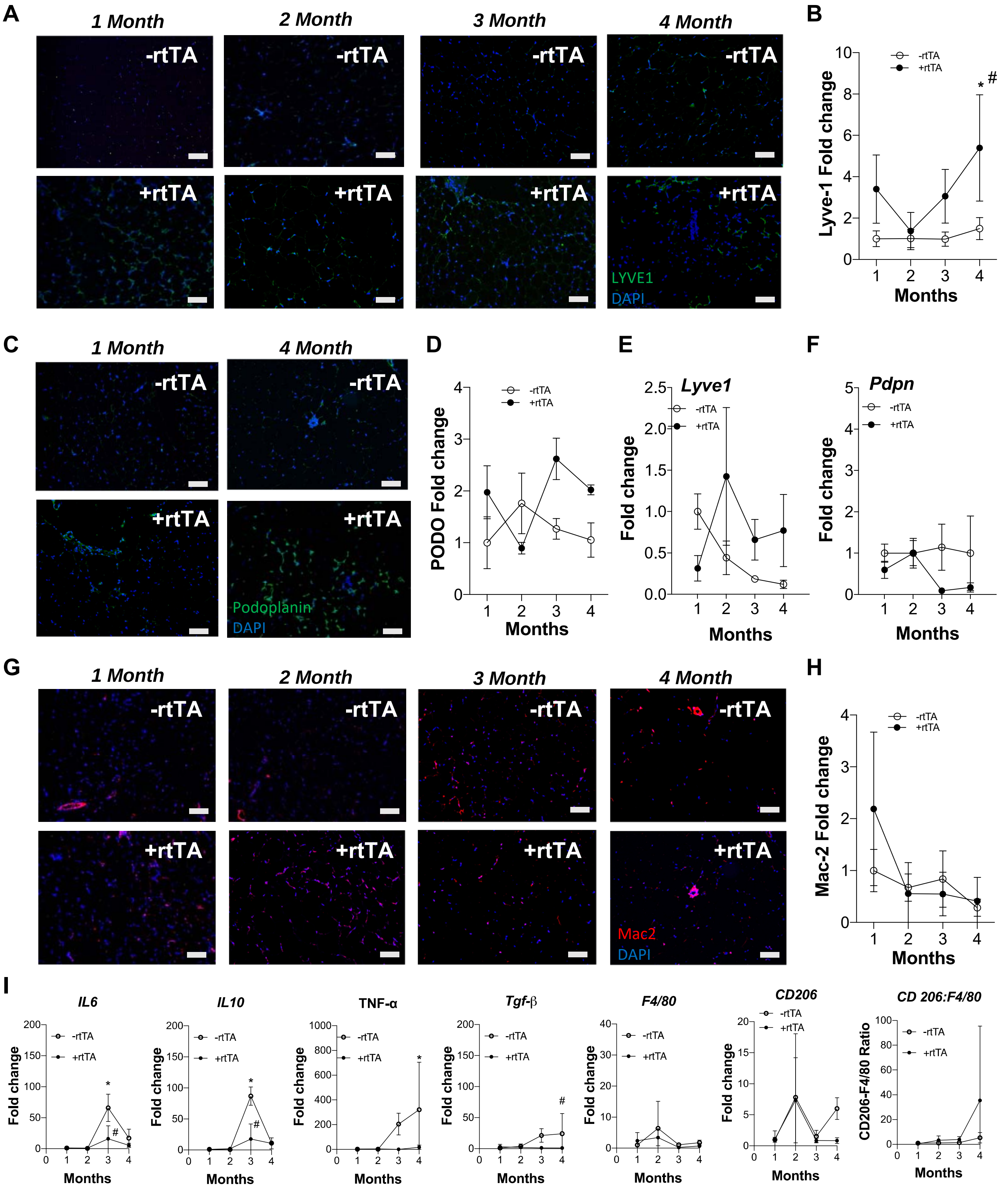
A
1 Month
2 Month
3 Month
4 Month
-rtTA
+rtTA
LYVE1
DAPI
B
-rtTA
+rtTA
Lyve-1 Fold change
Months
C
1 Month
4 Month
-rtTA
+rtTA
Podoplanin
DAPI
D
PODO Fold change
Months
E
Lyve1
Fold change
Months
F
Pdpn
Fold change
Months
G
1 Month
2 Month
3 Month
4 Month
-rtTA
+rtTA
Mac2
DAPI
H
Mac-2 Fold change
Months
I
IL6
IL10
TNF-α
Tgf-β
F4/80
CD206
CD 206:F4/80
Fold change
CD206-F4/80 Ratio
Months

Supplementary Figure 1: Subcutaneous inguinal adipose lymphatics and inflammation in chow fed male Adipo-VD mice.

A: LYVE1 (green) immunofluorescence of lymphatic structures in male –rtTA and +rtTA subcutaneous inguinal adipose following 1, 2, 3, and 4-month chow diet feeding. B: LYVE1 pixel area fold change comparison between –rtTA vs +rtTA mouse inguinal depots quantified from random imaging and all values normalized to –rtTA tissues at 1 month. C: Podoplanin immunofluorescence (green) of lymphatic structures in male –rtTA and +rtTA inguinal adipose depot following 1 and 4-month chow diet feeding D: Podoplanin pixel area fold change comparison between –rtTA vs +rtTA inguinal depot quantified from random imaging and all values normalized to –rtTA tissues at 1 month. E, F: QPCR time course relative expression of *Lyve1* and *Pdpn* between –rtTA and +rtTA inguinal depot normalized to untreated control mouse adipose. G: Macrophage $Mac2^{+}$ crown like structures (red) in –rtTA and +rtTA male subcutaneous inguinal adipose depot following 1, 2, 3, and 4-month chow diet feeding. H: Mac2+ pixel area fold change comparison between –rtTA vs +rtTA inguinal depot quantified from random imaging and all values normalized to –rtTA tissues at 1 month. I: QPCR immune profile of *IL6, IL10, TNF-α, TGF-β, CD206, F4/80* and ratio of *CD206:F4/80*-fold change across the time course normalized to untreated control mouse adipose. Images A-G, blue=DAPI and scale bars=20μm. (B, H) n = 8,8. (D, E, F, I) n=5,5. $^{*}P < 0.05$ versus –rtTA; #P<0.05 versus 1 month.

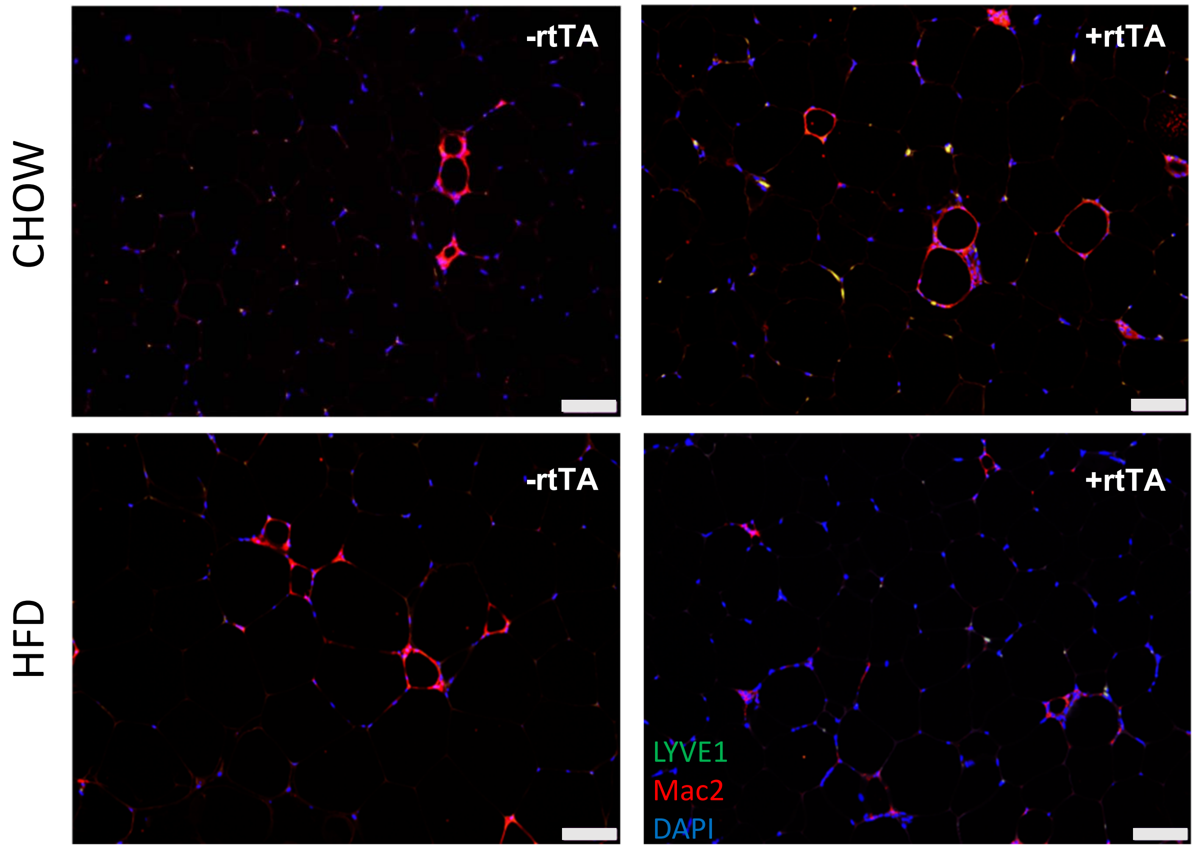

Supplementary Figure 2: Gonadal adipose tissue lymphatics and crown-like structures.

Representative images of LYVE1 (green) and Mac2 (red) immunofluorescence find no lymphatics within the gonadal adipose tissue depot of chow and HFD –rtTA and +rtTA mice. Blue=DAPI and scale bars=20μm.

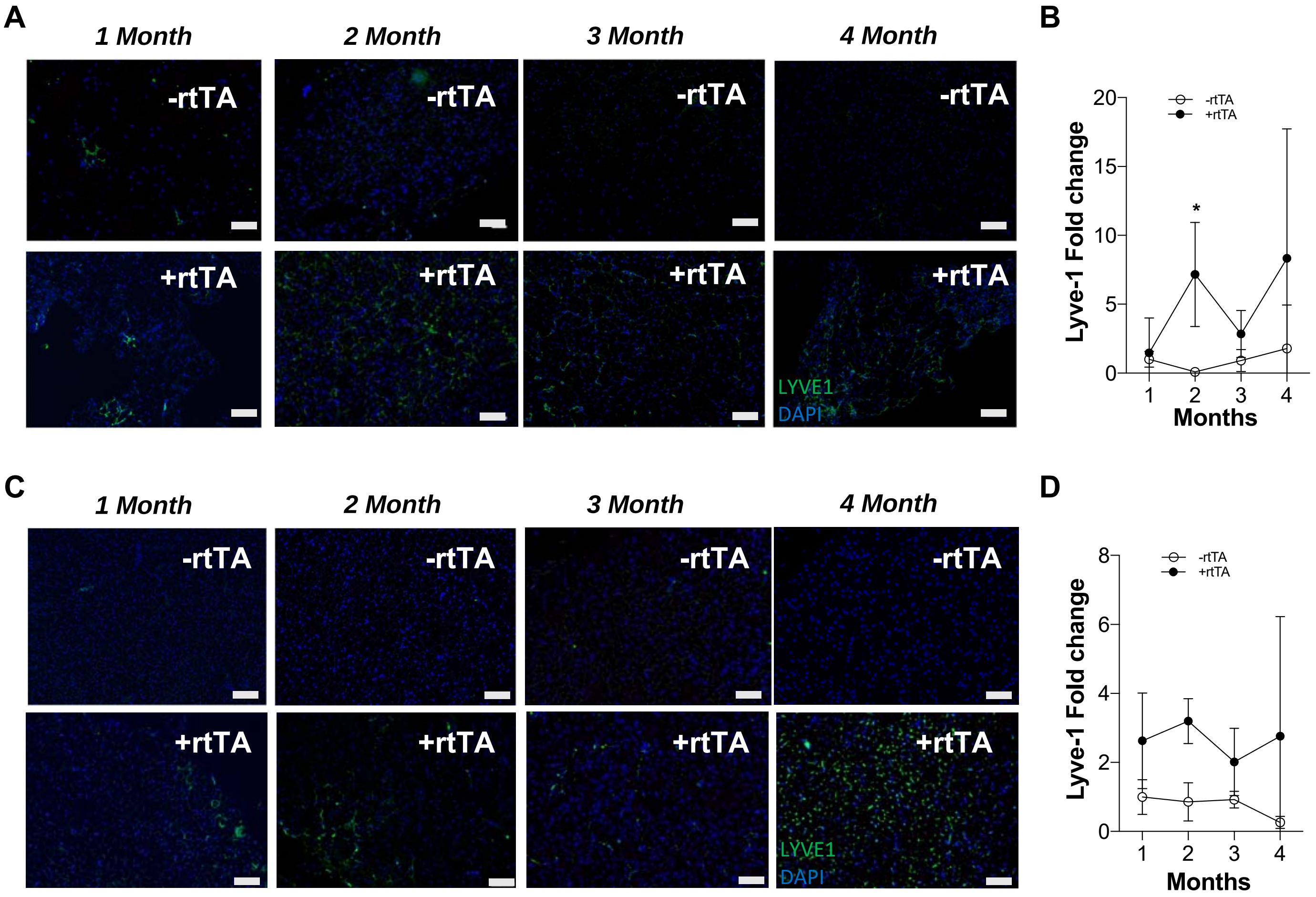

Supplementary Figure 3: Interscapular brown adipose lymphatic expansion in high fat diet and chow fed male Adipo-VD mice.

A: LYVE1 (green) immunofluorescence of lymphatic structures in male –rtTA and +rtTA interscapular brown adipose tissue following 1, 2, 3, and 4-month high fat diet feeding. B: LYVE1 pixel area fold change comparison between high fat diet fed –rtTA vs +rtTA mouse brown adipose depots quantified from random imaging and all values normalized to –rtTA tissues at 1 month. C: LYVE1 (green) immunofluorescence of lymphatic structures in male –rtTA and +rtTA interscapular brown adipose tissue following 1, 2, 3, and 4-month chow diet feeding. D: LYVE1 pixel area fold change comparison between chow fed –rtTA vs +rtTA mouse brown adipose depots quantified from random imaging and all values normalized to –rtTA tissues at 1 month. Images A, C blue=DAPI and scale bars=20µm (B, D) $n = 8$. $^{*}P < 0.05$ versus −rtTA.

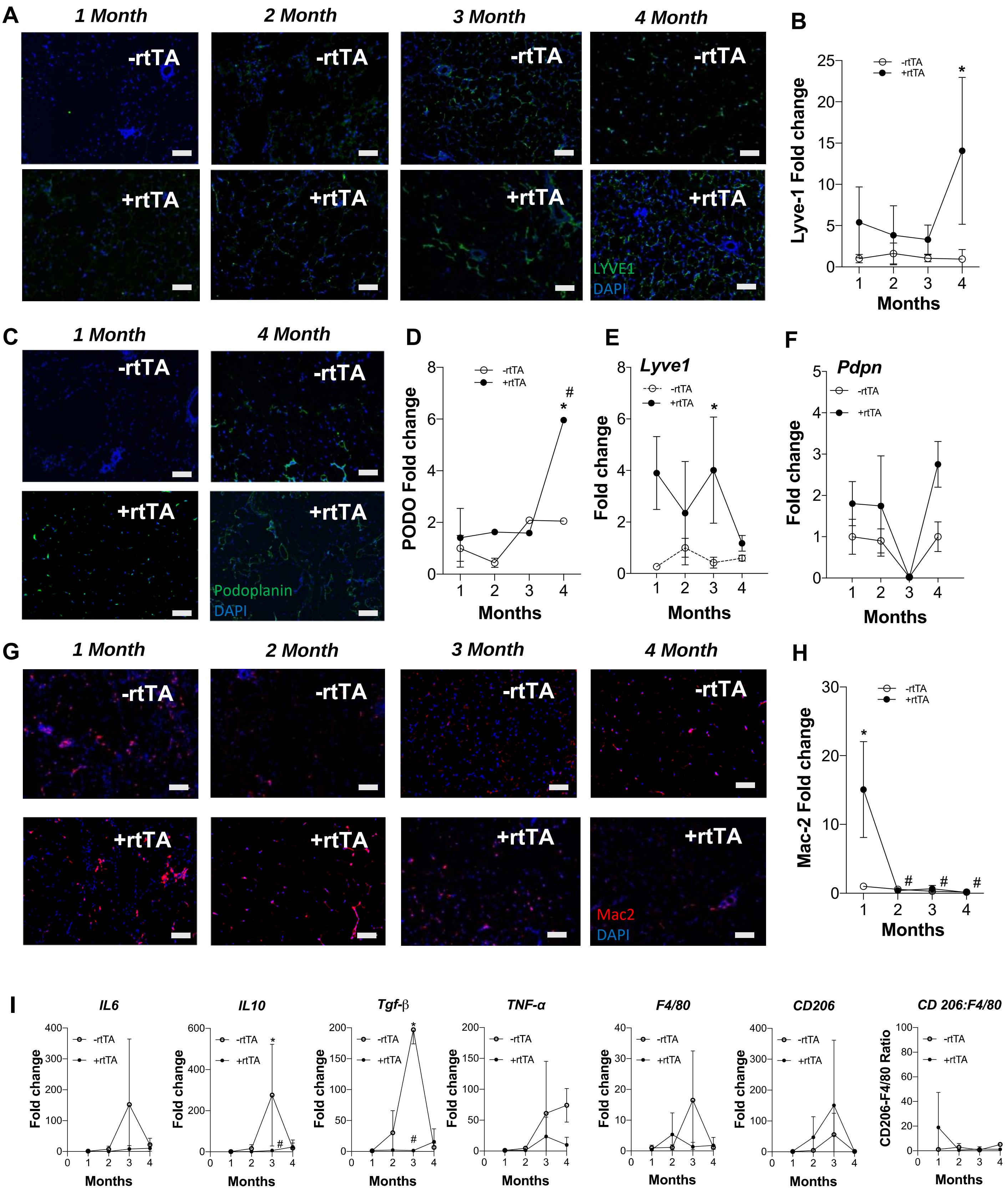
A
1 Month
2 Month
3 Month
4 Month
-rtTA
+rtTA
LYVE1
DAPI
B
Lyve-1 Fold change
Months
C
1 Month
4 Month
Podoplanin
DAPI
D
PODO Fold change
E
Lyve1
F
Pdpn
Fold change
G
Mac2
H
Mac-2 Fold change
I
IL6
IL10
Tgf-β
TNF-α
F4/80
CD206
CD 206:F4/80
CD206-F4/80 Ratio

Supplementary Figure 4: Subcutaneous inguinal adipose lymphatics and inflammation in chow fed female Adipo-VD mice.

A: LYVE1 (green) immunofluorescence of lymphatic structures in female –rtTA and +rtTA subcutaneous inguinal adipose following 1, 2, 3, and 4-month chow diet feeding. B: LYVE1 pixel area fold change comparison between –rtTA vs +rtTA mouse inguinal depots quantified from random imaging and all values normalized to –rtTA tissues at 1 month. C: Podoplanin immunofluorescence (green) of lymphatic structures in female –rtTA and +rtTA inguinal adipose depot following 1 and 4-month chow diet feeding D: Podoplanin pixel area fold change comparison between –rtTA vs +rtTA inguinal depot quantified from random imaging and all values normalized to –rtTA tissues at 1 month. E, F: QPCR time course relative expression of *Lyve1* and *Pdpn* between –rtTA and +rtTA inguinal depot normalized to untreated control mouse adipose. G: Macrophage $Mac2^{+}$ crown like structures (red) in –rtTA and +rtTA female subcutaneous inguinal adipose depot following 1, 2, 3, and 4-month chow diet feeding. H: Mac2+ pixel area fold change comparison between –rtTA vs +rtTA inguinal depot quantified from random imaging and all values normalized to –rtTA tissues at 1 month. I: QPCR immune profile of *IL6, IL10, TNF-α, TGF-β, CD206, F4/80* and ratio of *CD206:F4/80*-fold change across the time course normalized to untreated control mouse adipose. Images A-G, blue=DAPI and scale bars=20µm. (B, H) n = 8,8. (D, E, F, I) n=5,5. $^{*}P < 0.05$ versus –rtTA; #P<0.05 versus 1 month.

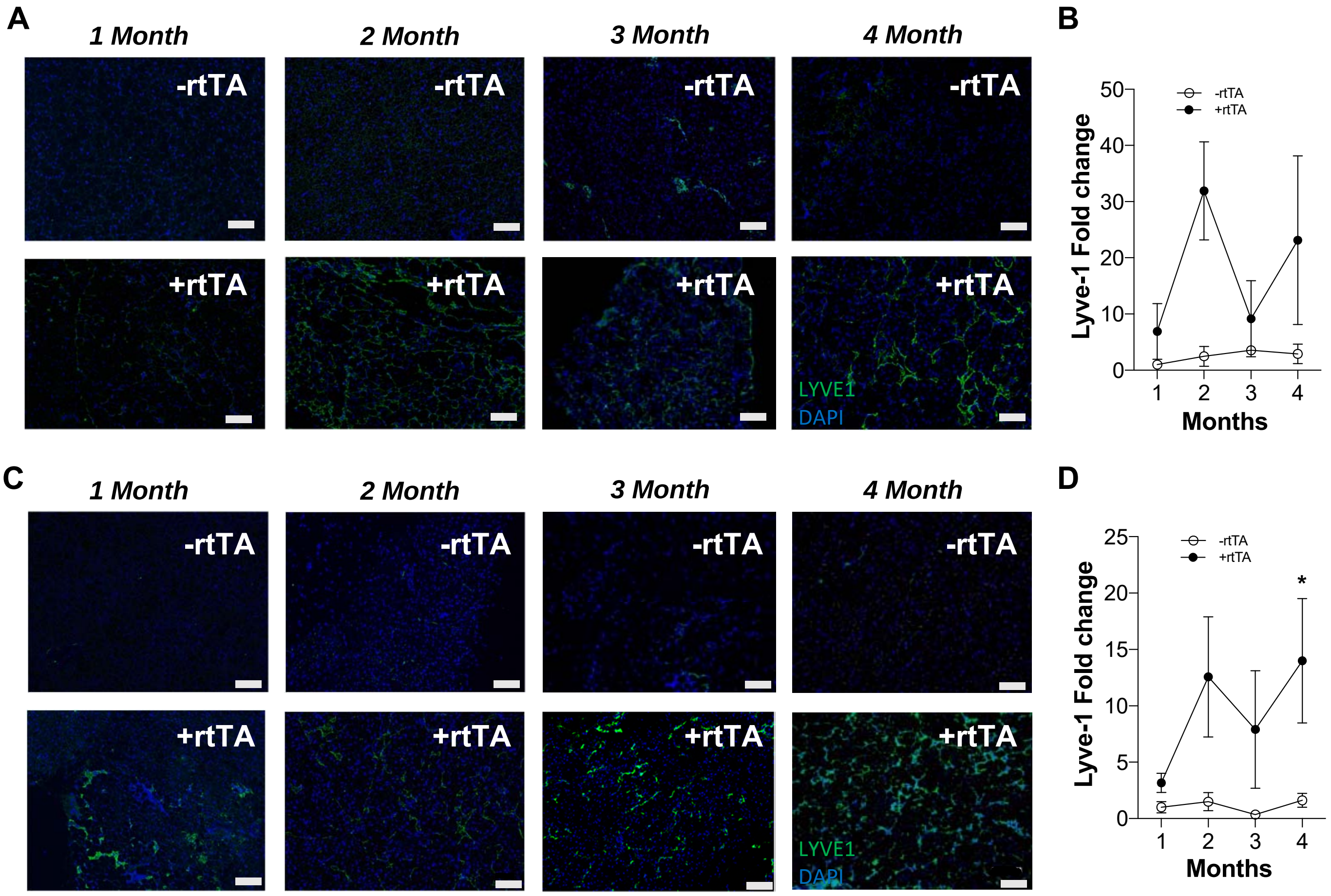

Supplementary Figure 5: Interscapular brown adipose lymphatic expansion in high fat diet and chow fed female Adipo-VD mice.

A: LYVE1 (green) immunofluorescence of lymphatic structures in female –rtTA and +rtTA interscapular brown adipose tissue following 1, 2, 3, and 4-month high fat diet feeding. B: LYVE1 pixel area fold change comparison between high fat diet fed –rtTA vs +rtTA mouse brown adipose depots quantified from random imaging and all values normalized to –rtTA tissues at 1 month. C: LYVE1 (green) immunofluorescence of lymphatic structures in female –rtTA and +rtTA interscapular brown adipose tissue following 1, 2, 3, and 4-month chow diet feeding. D: LYVE1 pixel area fold change comparison between chow fed –rtTA vs +rtTA mouse brown adipose depots quantified from random imaging and all values normalized to –rtTA tissues at 1 month. Images A, C blue=DAPI and scale bars=20µm (B, D) $n = 8$. $^*P < 0.05$ versus −rtTA.
